# Chronic Oxidative Stress and Stress Granule Formation in UBQLN2 ALS Neurons: Insights into Neuronal Degeneration and Potential Therapeutic Targets

**DOI:** 10.3390/ijms252413448

**Published:** 2024-12-15

**Authors:** Ao Gu, Yiti Zhang, Jianfeng He, Mingri Zhao, Lingjie Ding, Wanxi Liu, Jianing Xiao, Jiali Huang, Mujun Liu, Xionghao Liu

**Affiliations:** 1 MOE Key Lab of Rare Pediatric Diseases & Hunan Key Laboratory of Medical Genetics of the School of Life Sciences, Central South University, Changsha 410017, China; guao@sklmg.edu.cn (A.G.); zhangyiti@sklmg.edu.cn (Y.Z.); hejianfeng@sklmg.edu.cn (J.H.); zhaomingri@sklmg.edu.cn (M.Z.);; 2 Department of Cell Biology, School of Life Sciences, Central South University, Changsha 410017, China; 3 Hunan Key Laboratory of Basic and Applied Hematology, Central South University, Changsha 410017, China; 4 Hunan Key Laboratory of Animal Model for Human Diseases, Central South University, Changsha 410017, China

**Keywords:** neurodegenerative diseases, oxidative stress, UBQLN2, ALS, stress granule, motor neurons

## Abstract

The pathogenesis of neurodegenerative diseases results from the interplay between genetic and environmental factors. Aging and chronic oxidative stress are critical contributors to neurodegeneration. UBQLN2, a ubiquitin-related protein, aids in protein degradation and protects against oxidative stress. In ALS neurons harboring UBQLN2 mutations, oxidative stress accelerates pathological changes, yet the precise mechanisms remain unclear. Using induced motor neurons (iMNs) derived from UBQLN2 P497H iPSCs, we observed ALS-like phenotypes, including TDP-43 mislocalization, increased cell death, and reduced viability. Sodium arsenite (SA)-induced oxidative stress triggered stress granule formation, while autophagy dysfunction exacerbated neuronal degeneration. CHX and bosutinib treatments reduced ubiquitinated protein accumulation and alleviated degeneration, highlighting potential therapeutic pathways. These findings emphasize the role of chronic oxidative stress and stress granule formation in UBQLN2 ALS, offering insights into novel therapeutic targets.

## 1. Introduction

Amyotrophic lateral sclerosis (ALS) is a progressive neurodegenerative disease characterized by the degeneration of motor neurons in the brain and spinal cord, leading to muscle weakness, atrophy, and eventual death. The exact etiology of ALS is complex and multifactorial, involving genetic, environmental, and molecular factors [1,2,3]. Among genetic factors, mutations in genes, including SOD1, TDP-43, FUS, C9orf72, and UBQLN2, have been implicated in the pathogenesis of ALS [4,5,6,7,8].

Oxidative stress, which increases with aging and is caused by an imbalance between the production of reactive oxygen species (ROS) and the body’s antioxidant defenses, is a key factor in many neurodegenerative diseases, including ALS [9,10,11,12,13,14]. Protein misfolding and aggregation are critical mechanisms in ALS pathogenesis, with mutations in genes such as UBQLN2, SOD1, TDP-43, and C9orf72 leading to protein misfolding and aggregation, thereby causing cellular stress and toxicity [15,16,17,18,19]. Age-related oxidative stress damages proteins, lipids, and DNA, exacerbating protein misfolding and aggregation, which contributes to neuronal dysfunction and death [13,20].

UBQLN2 (ubiquitin-like protein 2) is a member of the ubiquitin family and plays a crucial role in linking the ubiquitin-proteasome system (UPS) with autophagy [21,22,23]. These pathways are essential for maintaining cellular homeostasis by preventing the accumulation of potentially toxic proteins. Mutations in UBQLN2 impair these pathways, leading to the accumulation of protein aggregates [18,19]. These aggregates are not only byproducts of cellular dysfunction, but also actively contribute to cytotoxicity, exacerbating neurodegenerative pathology. UBQLN2 is recognized as a significant pathogenic gene in ALS, where mutations in UBQLN2 lead to X-linked ALS/FTD [21]. Moreover, UBQLN2 aggregate accumulation has been observed in many ALS cases without UBQLN2 mutations [21].

The prevailing view is that UBQLN2-linked ALS/FTD is driven by a combination of both loss-of-function and gain-of-function toxicity [24]. Interestingly, while abnormal protein aggregation has been linked to ALS pathology in many UBQLN2 animal models, UBQLN2 knockout (KO) rats do not exhibit any pathological changes [25,26,27]. Similarly, UBQLN2 KO mice do not show signs of neurodegeneration [28]. Recent studies involving in vitro liquid–liquid phase separation (LLPS) have shown that UBQLN2 mutation enhances its oligomerization tendency and co-separation with various ubiquitinated proteins, thereby resisting proteasomal degradation [29]. Our previous studies have demonstrated that UBQLN2 overexpression leads to the formation of aggregated droplets within cells, while endogenous UBQLN2 is typically diffusely distributed [30]. These findings suggest that the aggregation of UBQLN2 may play a more critical role in ALS than the loss of UBQLN2 function. Our earlier research also revealed that UBQLN2 co-localizes with stress granules (SGs) formed under oxidative stress, undergoing liquid–liquid phase separation [30,31]. Simultaneously, UBQLN2 mutations delayed the clearance of UBQLN2-positive aggregates in cells [31]. These results indicate that oxidative stress may play a crucial role in the pathology of UBQLN2-associated ALS.

SGs are dynamic, reversible protein–RNA aggregates that form under stress conditions, such as oxidative stress, heat shock, and energy deprivation, to protect cellular functions. However, their persistent or aberrant accumulation is associated with the pathogenesis of neurodegenerative diseases. The pathological aggregation of TDP-43 in neurons is a hallmark of ALS, and chronic oxidative stress has been shown to localize TDP-43 within SGs [4,32]. Over the past decade, many ALS-associated proteins, including UBQLN2, TDP-43, FUS, and C9orf72, have been identified as SG components, and it is hypothesized that SG formation may trigger neurodegenerative processes by sequestering critical RNA-binding proteins (RBPs) and factors [6,30,32,33]. Our previous research found that the ALS-causing UBQLN2 P497H mutation promotes SG formation and renders cells more sensitive to TDP-43 cytoplasmic mislocalization induced by oxidative stress [30,31].

The relationship between oxidative stress and neuropathology in UBQLN2 ALS neurons remains unknown. In FUS mutant mouse models, the mutated FUS protein mislocalizes to SGs, leading to an upregulation of ubiquitin proteins [33]. Both autophagy and the ubiquitin-proteasome system are involved in the clearance of SGs [34]. Given that UBQLN2 is involved in SG formation, the disruption of the ubiquitin-proteasome system and autophagy due to UBQLN2 mutations may be related to SG formation. Recent studies have indicated that oxidative stress-related SGs play a significant role in programmed cell death and are essential for the activation of necrotic cell death [33,35].

To elucidate whether oxidative stress may contribute to UBQLN2 ALS neuropathology through SG-associated abnormal protein aggregates, we established an NIL-iMN model. Using sodium arsenite, we found that chronic oxidative stress induced axonal swelling, impaired axonal transport, and caused cell death, all indicative of neuropathology. Like UBQLN2 mutations, oxidative stress led to increased SG formation, progressive autophagy abnormalities, increased apoptosis, and axonal degeneration with impaired axonal transport. Inhibition of pathways related to oxidative stress-associated SG formation partially rescued neuronal degeneration and death. These results support the idea that abnormal protein accumulation contributes to ALS neuropathology, and suggest that oxidative stress plays a role in UBQLN2 mutation-induced neurotoxicity in ALS.

## 2. Results

### 2.1. In the NIL-iMN Model, UBQLN2 Mutant Neurons Exhibit TDP-43 Cytoplasmic Mislocalization and Other Neurodegenerative Pathologies

**Figure 1:**
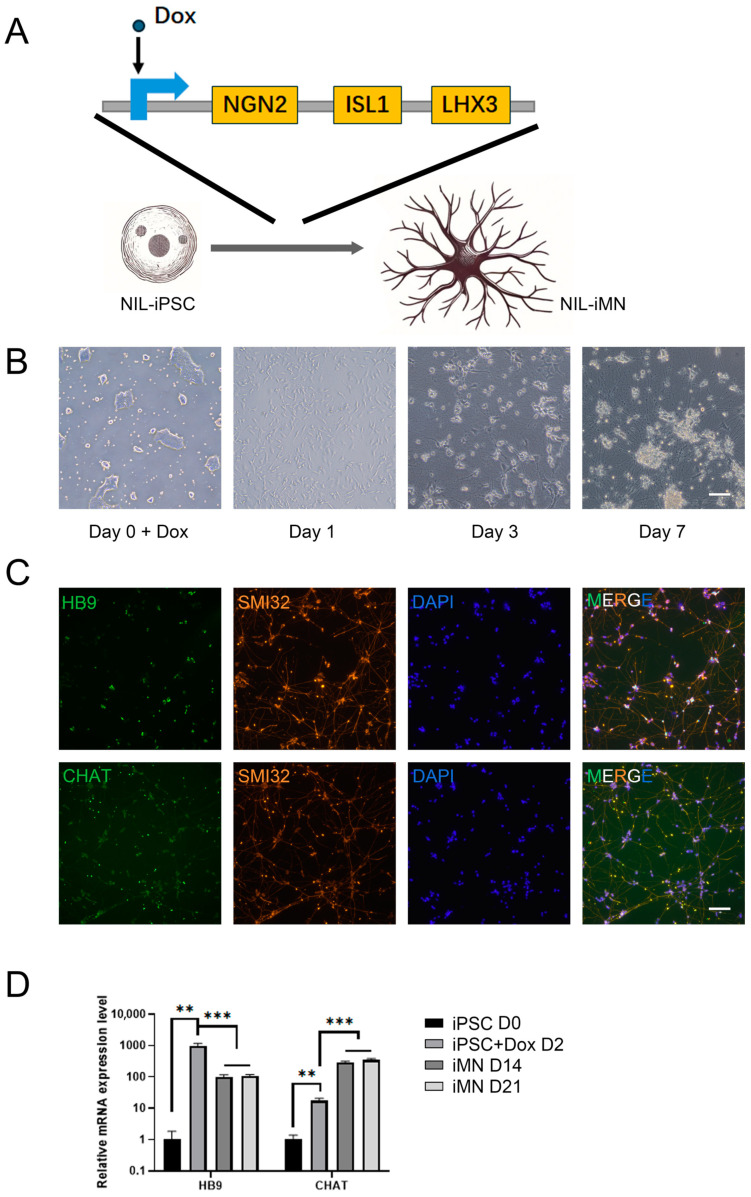
Establishment and Characterization of P497H-NIL-iMN. (**A**) Schematic diagram of the differentiation principle: Dox induction of NIL gene expression drives the differentiation of NIL-iPSCs into NIL-iMNs; (**B**) P497H-NIL-iMN differentiation process: representative images from day 0, day 1, day 3, and day 7. Scale bar: 100 μm; (**C**) immunofluorescence detection of motor neuron markers in P497H-NIL-iMNs: cells at day 4 of differentiation were stained with anti-HB9, SMI32, and CHAT antibodies. Scale bar: 100 μm; (**D**) dynamic expression of motor neuron markers HB9 and CHAT in P497H-NIL-iMNs (*n* = 3): expression levels were measured in iPSCs/iMNs at day 0 (before Dox induction), and at days 2, 14, and 21 after differentiation. Data are means ± SEM. ** *p* < 0.01, *** *p* < 0.001. Statistics by one-way analysis of variance (ANOVA).

Specific transcription factors, Neurogenin2 (Ngn2), insulin gene enhancer 1 (Isl1), and LIM homeobox 3 (Lhx3), can induce rapid differentiation of human pluripotent stem cells into motor neurons [36,37]. In this study, we used the CRISPR system to introduce a conditionally expressed gene cluster NIL (NGN2, ISL1, LHX3) into the safe harbor locus of healthy iPSCs and UBQLN2 mutant iPSCs at the CLYBL locus (Figure 1A). Clones were confirmed by fluorescence, flow cytometry, and PCR, and sequencing ruled out CRISPR off-target effects (Figure S1, Table S1). This resulted in the generation of normal control lines WT-NIL-iPSC1, WT-NIL-iPSC2 (WT-NIL-iPSC1 and WT-NIL-iPSC2 are two distinct healthy human iPSC lines), and a line carrying the UBQLN2 P497H mutation, named P497H-NIL-iPSC (P497H-NIL-iPSC shares the same genetic background as WT-NIL-iPSC2, as they both originate from the same iPSC line).

Using 3 µg/mL Dox to conditionally express NIL, we induced iPSC differentiation into motor neurons through a two-stage culture process, resulting in HB9 and CHAT-positive motor neurons, designated as WT-NIL-iMN1, WT-NIL-iMN2, and P497H-NIL-iMN (unless otherwise specified, WT-NIL-iMN/WT-iMN in the following text refers to WT-NIL-iMN2). Figure 1B illustrates representative images from the differentiation process, showing that by the first day of differentiation, cells began extending neurites, by day three, extensive neurite networks were formed, and by day seven, dense neuronal networks were observed. Immunofluorescence confirmed that the differentiated cells were positive for motor neuron markers HB9, CHAT, and SMI32 (Figure 1C). Quantitative PCR demonstrated that this differentiation rapidly increased HB9 and CHAT expression, which remained stable for up to 21 days (Figure 1D).

**Figure 2:**
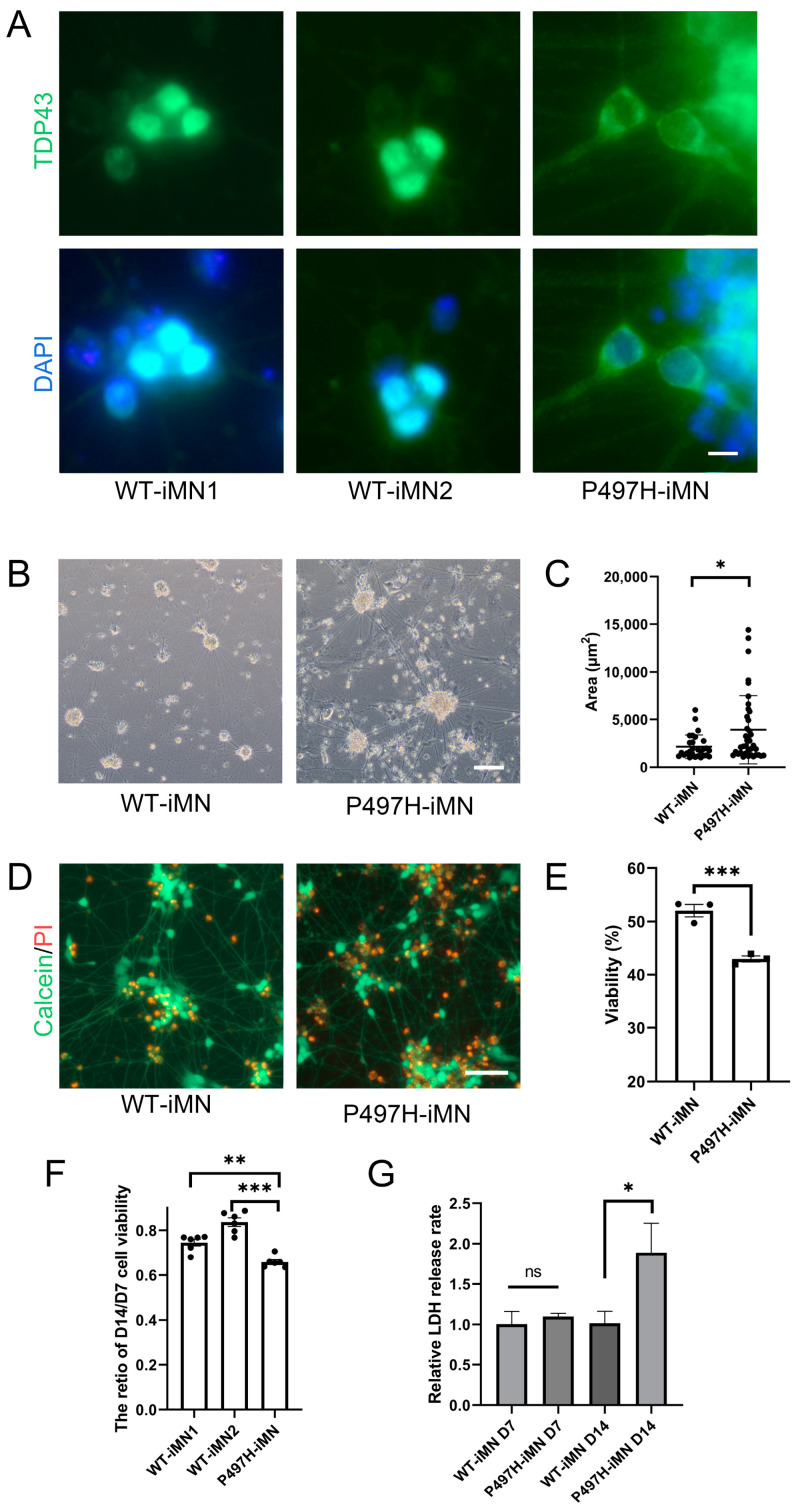
ALS-Related pathology in UBQLN2 mutant motor neurons. (**A**) Immunofluorescence analysis of TDP-43 localization in day 14 iMNs: under physiological conditions, TDP-43 is localized in the nucleus, while pathological conditions show TDP-43 mislocalization to the cytoplasm. Arrows indicate TDP-43 mislocalization in P497H-NIL-iMNs. Scale bar: 50 μm; (**B**,**C**) representative images and statistical analysis of neuronal soma size in WT-iMN2 and P497H-iMN at day 21 of differentiation (*n* > 25). Scale bar: 100 μm; (**D**,**E**) Calcein/PI analysis of cell viability in WT-iMN2 and P497H-iMN (*n* = 3): Calcein AM generates strong green fluorescence in live cells containing esterases, while PI stains the nuclei of cells with compromised membrane integrity. Scale bar: 100 μm; (**F**) ATP cell viability analysis of iMNs at days 14 and 7 (*n* ≥ 5): iMNs were differentiated in two 96-well plates, and ATP levels were measured at days 7 and 14 to assess the rate of decline in cell viability; (**G**) LDH release rate analysis: media and cell lysates from WT-iMN2 and P497H-iMN were collected at the indicated time points after complete media replacement 3 days prior, and LDH release rates were calculated (*n* ≥ 5). Data are means ± SEM. ns means “no significance”, * *p* < 0.05, ** *p* < 0.01, *** *p* < 0.001. Statistics by Mann–Whitney tests in (**C**), Student’s *t* tests in (**E**,**G**), and one-way ANOVA in (**F**).

Cytoplasmic mislocalization of TDP-43 is a hallmark event in ALS [38,39]. Immunofluorescence analysis revealed a significant increase of TDP-43 mislocalization in UBQLN2 mutant neurons, whereas almost no TDP-43 mislocalization was observed in control healthy neurons (Figure 2A). Neuronal death, a characteristic feature of late-stage ALS pathology, was also evident. iMNs are adherent cells, and their death is accompanied by a detachment process. During differentiation, we observed that UBQLN2 P497H-NIL-iMNs exhibited larger cellular aggregates (Figure 2B,C and Figure S2), potentially related to cell death. To confirm this possibility, we performed Calcein/PI assays to assess cell viability and cytotoxicity (Figure 2D,E). We found that the progressively enlarging cell aggregates contained many PI-positive cells, indicating an increase in apoptosis in P497H-iMNs. Further ATP viability assays (Figure 2F) showed a more rapid decline in the viability of P497H-iMNs. Since LDH release is a marker of cellular senescence, we analyzed LDH release levels (Figure 2G) in neurons and found that P497H-iMNs released more LDH in day 14. Our findings suggest that reduced cell viability and increased cell death in P497H-iMNs are associated with ALS-like neurodegenerative pathology, marked by TDP-43 mislocalization.

### 2.2. Oxidative Stress Contributes to Neurodegeneration and Protein Aggregation in P497H-iMN

**Figure 3:**
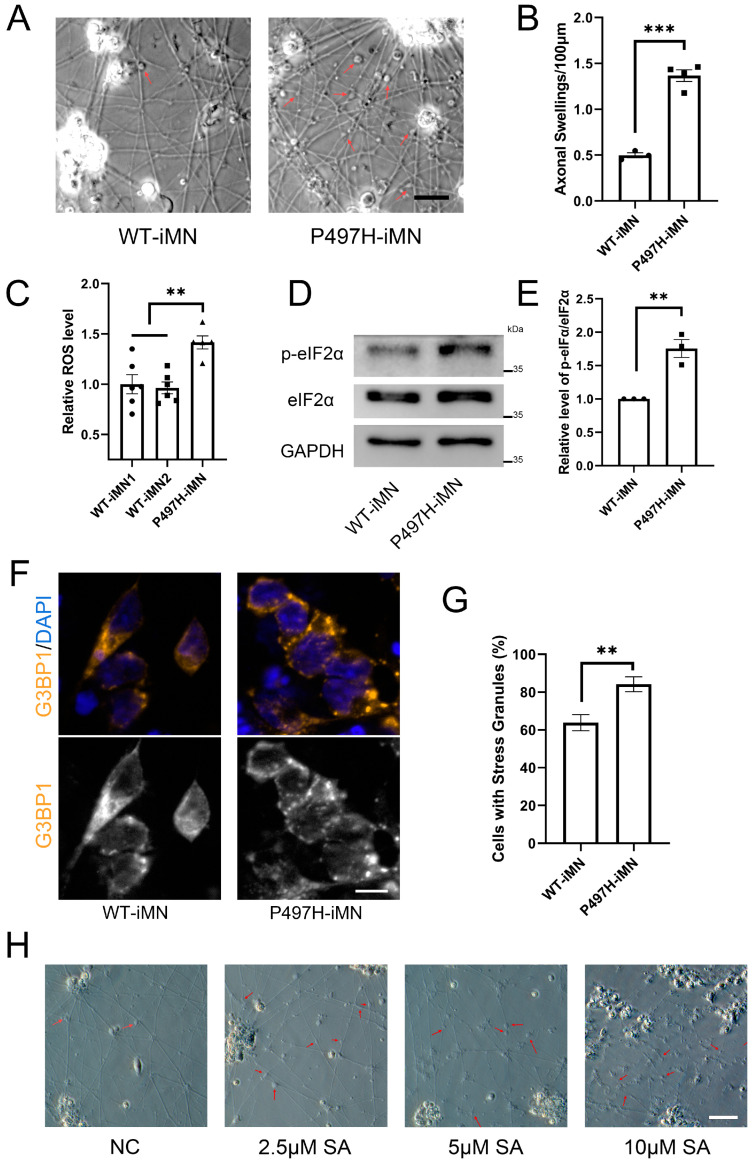
Oxidative stress and neuropathology in P497H-iMNs. (**A**,**B**) Examination of axonal damage in P497H-iMNs: iMN morphology was recorded on day 14 using microscopy. Arrows indicate axonal swelling. Scale bar: 20 μm. Quantification of axonal swelling was performed by counting the number of swollen vesicles per 100 µm of axon across at least 3 fields, with a minimum of 5 axonal segments per field. (**C**) ROS levels in iMNs were measured using DCFH-DA, with at least 4 replicates per group. (**D**,**E**) Western blot analysis of p-eIF2α levels in iMN day 14 lysates, with quantification (*n* = 3). (**F**,**G**) Immunofluorescence detection and quantification of stress granules (SGs) in iMNs: cells were treated with 0.5 mM sodium arsenite (SA) for 45 min, and SGs were detected using G3BP1 as a marker. Scale bar: 10 μm. (**H**) Assessment of chronic oxidative stress on axonal pathology: iMNs were treated with varying concentrations of SA on day 7. Axon morphology was recorded after 48 h using light microscopy. Arrows indicate axonal swelling/fragmentation. Scale bar: 50 μm. Data are means ± SEM. ** *p* < 0.01, *** *p* < 0.001. Statistics by Student’s *t* test in (**B**,**E**,**G**) and one-way ANOVA in (**C**).

Neurodegeneration is a hallmark of neurodegenerative diseases, including amyotrophic lateral sclerosis (ALS). Axonal degeneration, characterized by axonal swelling, fragmentation, and eventual neuronal death, plays a crucial role in neurodegeneration. Through microscopic examination of induced motor neurons (iMNs), we observed a significantly higher incidence of axonal swelling in P497H-iMNs compared to WT-iMNs (Figure 3A,B). Chronic oxidative stress has been implicated in neuronal degeneration in ALS models with mutations in SOD1 and FUS [40,41]. To determine whether oxidative stress dysregulation occurs in motor neurons harboring the UBQLN2 mutation, we measured reactive oxygen species (ROS) levels in iMNs using the fluorescent probe DCFH-DA. The results revealed a marked elevation in ROS levels in P497H-iMNs compared to healthy controls (Figure 3C).

Given that ROS may trigger cellular stress responses, we performed Western blot analysis on iMN lysates. The results demonstrated significantly elevated levels of phosphorylated eIF2α (p-eIF2α) in P497H-iMNs compared to WT-iMNs (Figure 3D,E). p-eIF2α is a key marker of the integrated stress response, which inhibits protein translation and promotes stress granule formation, a process linked to aberrant protein accumulation [42,43]. To investigate whether stress granule formation is altered in motor neurons with UBQLN2 mutations, we treated iMNs with sodium arsenite (SA), a known inducer of oxidative stress. Under physiological conditions, G3BP1 is diffusely distributed in the cytoplasm. Following stress treatment, G3BP1 aggregates in the cytoplasm, reflecting the formation of stress granules. Immunofluorescence analysis showed a higher proportion of stress granule-positive cells in P497H-iMNs compared to WT-iMNs after SA treatment (Figure 3F,G). This observation is consistent with our previous findings in HeLa and 293T cell lines, suggesting that UBQLN2 mutations accelerate stress granule formation, which reflects abnormal protein aggregation within the cells.

To further explore the role of oxidative stress in P497H-associated axonal swelling, we treated iMNs with increasing concentrations of SA. Microscopic analysis revealed that axonal degeneration worsened with higher SA concentrations (Figure 3H). At 2.5 µM SA, there was a marked increase in axonal swelling compared to negative controls. At 5 µM SA, axonal fragmentation was evident, and at 10 µM, extensive axonal fragmentation and neuronal death were observed.

The findings reveal elevated ROS levels and abnormal protein aggregation in UBQLN2 mutant motor neurons. Oxidative stress can lead to axonal swelling, mirroring the effects of UBQLN2 mutations.

**Figure 4:**
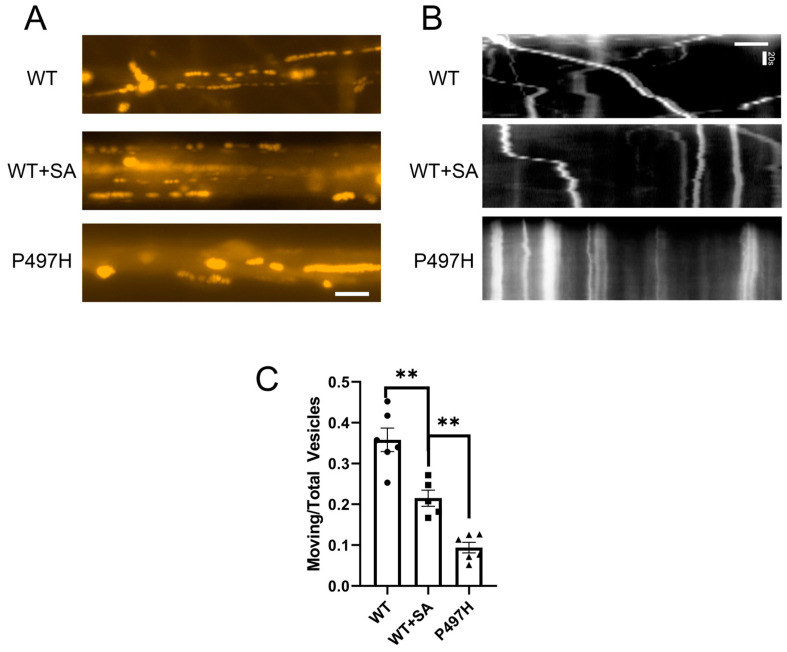
Impaired axonal lysosomal transport in neurons due to oxidative stress and UBQLN2 mutation. (**A**) Lysosomal labeling using LysoTracker-Red: maximum intensity projection visualized organelle movement trajectories within axons. Continuous, long trajectories indicate moving lysosomes, while discrete points highlight stationary organelles. Scale bar: 10 μm; (**B**) kymograph analysis to establish space-time plots: inclined lines represent moving lysosomes, and vertical lines indicate stationary organelles. Scale bar: 10 μm; (**C**) statistical analysis of the proportion of motile lysosomes in motor neurons: lysosomal movement in axons was quantified, with vesicles moving more than 20 μm during the observation period defined as motile lysosomes. At least 5 fields per group were analyzed, with vesicle count *n* > 250. Data are means ± SEM. ** *p* < 0.01. Statistics by one-way ANOVA.

Axonal degeneration impairs axonal function, and axonal swelling may lead to disrupted axonal transport. Axonal swelling in Alzheimer’s disease is associated with lysosomal accumulation in axons [44]. Similarly, we observed that LysoTracker signals were concentrated at sites of axonal swelling in iMNs (Figure S3). We used LysoTracker to label lysosomes and assess axonal transport in motor neurons (Video S1). Maximum intensity projection and kymograph analysis revealed lysosomal movement in iMNs (Figure 4A,B). Quantitative analysis of lysosomal axonal transport showed that over 30% of vesicles were motile in WT-iMNs, whereas only about 10% were motile in P497H-iMNs. The results showed that in iMNs, the proportion of moving vesicles in UBQLN2 P497H mutant neurons decreased (Figure 4C), and axonal transport was impaired. Chronic oxidative stress induced by SA treatment also led to impaired axonal transport, with only about 20% of vesicles remaining motile, possibly due to defective autophagic degradation of oxidative stress-induced misfolded proteins. These findings indicate that oxidative stress contributes to axonal degeneration and the pathology of UBQLN2 P497H ALS neurons.

### 2.3. CHX Rescues SG Formation and Cell Death Induced by Oxidative Stress

**Figure 5:**
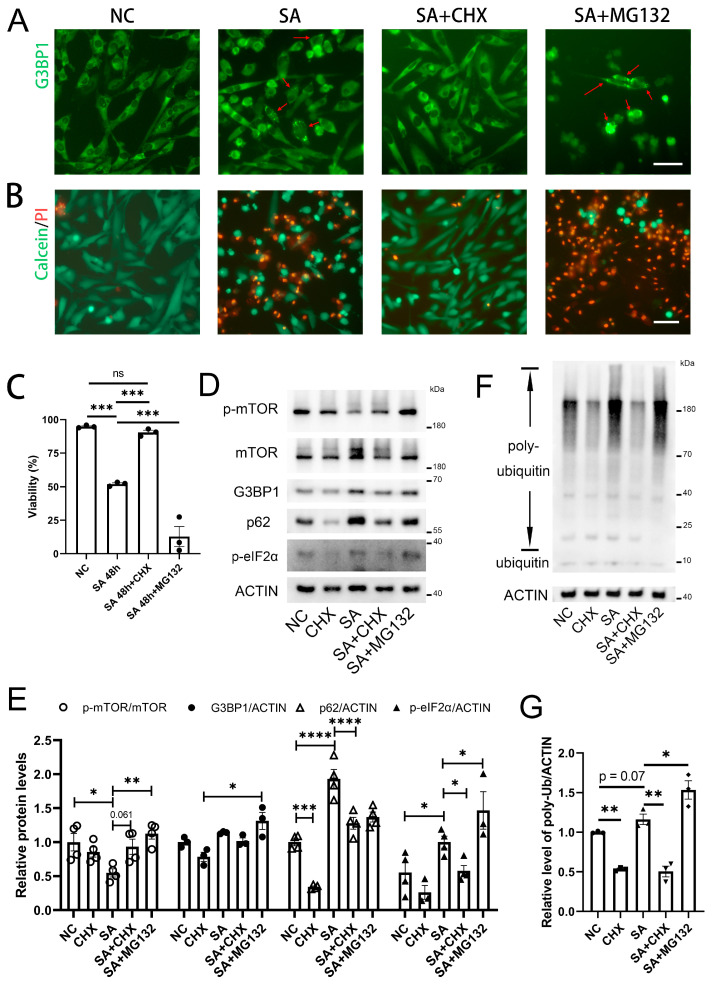
CHX inhibits SGs and cell death in HeLa cells. (**A**) HeLa cells expressing G3BP1-GFP were subjected to chronic oxidative stress induced by 20 μM SA and treated with 5 μM CHX and 5 μM MG132. G3BP1 aggregation indicating SGs was detected by fluorescence microscopy 48 h later. Arrows indicate SG-positive cells. Scale bar: 100 μm; (**B**,**C**) Calcein/PI analysis of cell viability in HeLa cells under the same treatment conditions, with statistical analysis of the results (*n* = 3). Scale bar: 20 μm; (**D**,**E**) Western blot analysis of HeLa cell lysates under the same treatment conditions (*n* ≥ 3); (**F**,**G**) Western blot and statistical analysis of poly-ubiquitin levels (*n* = 3). Data are means ± SEM. * *p* < 0.05, ** *p* < 0.01, *** *p* < 0.001, **** *p* < 0.0001; ns, nonsignificant. Statistics by one-way ANOVA in (**C**,**G**) and two-way ANOVA in (**E**).

Cellular senescence, as a form of chronic stress, is associated with the formation of SGs. SA is a well-studied oxidative stress inducer that promotes SG formation. Persistent SGs induced by chronic stress have been linked to cell death. To identify potential compounds that could mitigate cell death induced by chronic oxidative stress, we established a HeLa cell line with endogenous G3BP1 fused to GFP. Through preliminary screening of over a dozen compounds, we discovered that cycloheximide (CHX) exhibited promising effects. We treated HeLa cells with 20 µM SA for 24 h. Compared to the control group, chronic stress successfully induced SG formation and increased cell death (Figure 5A–C). Cycloheximide (CHX), a well-known translation inhibitor, has been observed to counteract apoptosis and support cell survival under various stress conditions, including oxidative stress [35]. Although CHX generally inhibits protein synthesis and can be harmful under normal physiological conditions, it demonstrates protective effects in stress scenarios. Here, we treated HeLa cells subjected to chronic oxidative stress with 5 μM CHX (Figure 5A–C). The results demonstrated that CHX effectively inhibited the formation of SGs, which are typically induced under oxidative stress conditions. Additionally, CHX treatment significantly rescued the cells from oxidative stress-induced death. In contrast, MG132 promoted SG formation and exacerbated cell death. MG132 is a proteasome inhibitor that promotes the accumulation of aberrant proteins by inhibiting proteasome function.

To investigate the potential mechanism by which cycloheximide (CHX) mitigates oxidative stress-induced stress granule formation and cell death, we conducted Western blot analysis on lysates from HeLa cells. The inactivation of mTOR and the phosphorylation of eIF2α disrupt the formation of the translation initiation complex, thereby inhibiting protein translation. Impaired translation initiation complexes can serve as seeds, facilitating stress granule formation with the assistance of G3BP1, which leads to abnormal protein accumulation and subsequent cell death. As expected, following sodium arsenite (SA) treatment, HeLa cells exhibited decreased levels of phosphorylated mTOR (p-mTOR), increased levels of phosphorylated eIF2α (p-eIF2α), and an accumulation of G3BP1, indicating proper stress granule formation (Figure 5D,E). When HeLa cells were treated with both CHX and SA, we observed an increase in p-mTOR levels, a decrease in p-eIF2α levels, and a reduction in G3BP1 protein levels compared to the SA-treatment group. Interestingly, the combined treatment with sodium arsenite (SA) and MG132 resulted in increased levels of p-mTOR compared to treatment with SA alone. Hu et al. proposed that the proteasome is involved in the clearance of mTOR, with MG132 inhibiting the proteasome and leading to the accumulation of both p-mTOR and mTOR, while CHX inhibits the synthesis of mTOR [45]. Additionally, the regulation of upstream signaling pathways of mTOR warrants further investigation. Considering that CHX alone did not significantly elevate p-mTOR levels, it is likely that CHX exerts its effects through pathways involving p-eIF2α and G3BP1.

Both the autophagy-lysosome system and the ubiquitin-proteasome system are involved in the clearance of abnormal proteins within cells. The accumulation of abnormal proteins promotes stress granule formation and accelerates cell death. p62 serves as a substrate for autophagy, while polyubiquitin reflects the levels of ubiquitinated substrates. Following SA treatment, we observed elevated levels of p62 and poly-ubiquitin, suggesting that abnormal proteins were not being effectively cleared (Figure 5D–G). However, CHX treatment led to a reduction in p62 and poly-ubiquitin levels, indicating that CHX may regulate both the autophagy and ubiquitin-proteasome systems, contributing to its resistance against oxidative stress.

### 2.4. CHX Rescues Neuronal Degeneration Induced by Oxidative Stress

**Figure 6:**
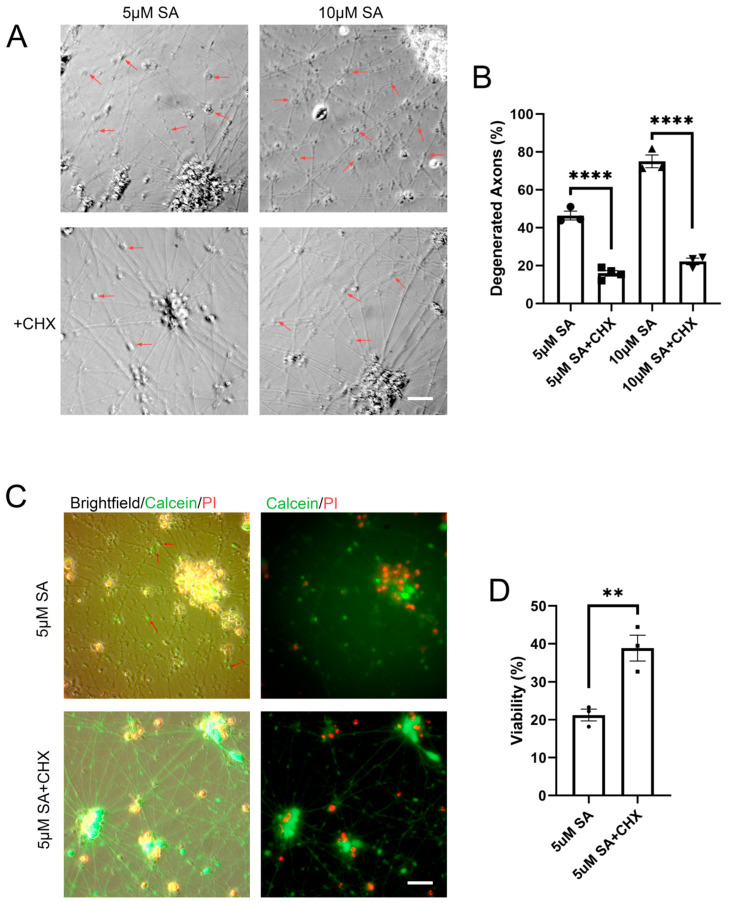
CHX rescues motor neuron degeneration. (**A**,**B**) iMNs at day 11 were treated with different concentrations of SA to induce oxidative stress, followed by treatment with 5 μM CHX. Neuronal morphology was examined 48 h later, and the ratio of degenerated axons was statistically analyzed (*n* ≥ 3). Scale bar: 25 μm; (**C**,**D**) Calcein/PI analysis of cell viability in iMNs 72 h after the same treatment, with statistical analysis (*n* = 3). Arrows indicate axonal degeneration. Scale bar: 25 μm. Data are means ± SEM. ** *p* < 0.01, **** *p* < 0.0001. Statistics by one-way ANOVA in (**B**), and Student’s *t* test in (**D**).

Oxidative stress can lead to axonal swelling and fragmentation in neurons. We sought to determine whether CHX could benefit motor neurons under chronic oxidative stress. To investigate this, neurons were treated with 5 µM CHX for 48 h under chronic oxidative stress, and axonal degeneration was observed using microscopy (Figure 6A). The results showed that 5 µM and 10 µM SA treatments induced axonal swelling and fragmentation, which were reduced following CHX treatment (Figure 6A,B). Additionally, Calcein/PI assays revealed that CHX significantly rescued cell death induced by oxidative stress (Figure 6C,D).

**Figure 7:**
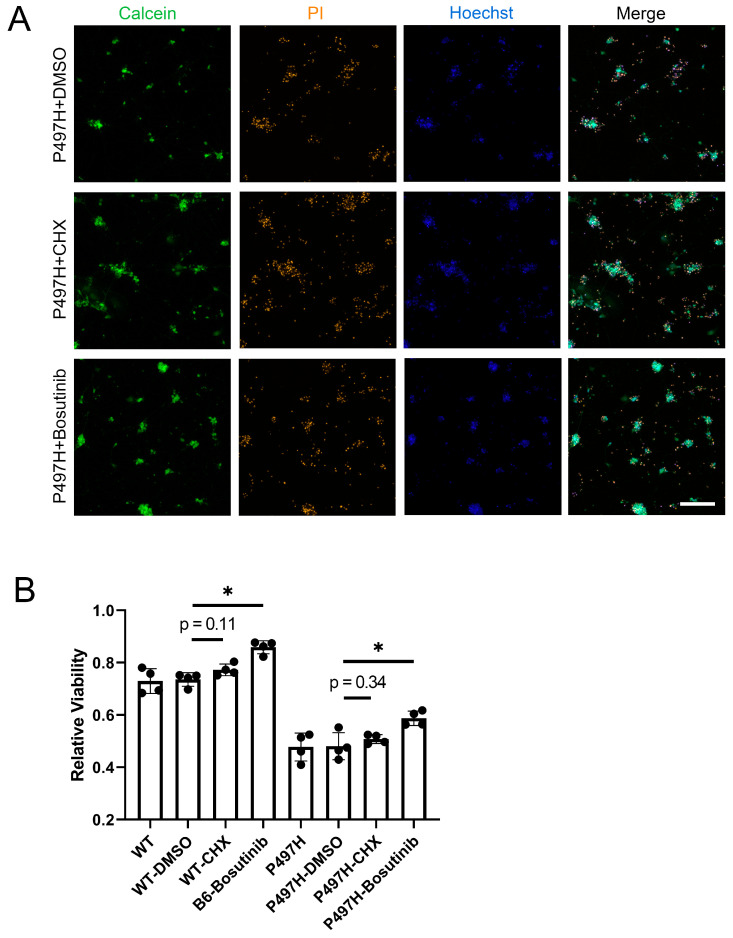
Effects of CHX and bosutinib on cell viability. (**A**) iMNs at day 7 were treated with 1 μM CHX and 1 μM bosutinib, and Calcein/PI analysis was performed and imaged at day 14. Scale bar: 100 μm; (**B**) statistical analysis of the Calcein/PI results (*n* = 4). Data are means ± SEM. * *p* < 0.05. Statistics by Student’s *t* test and one-way ANOVA in (**B**).

To further investigate whether CHX could play a role during neuronal aging, CHX was added at day 7 of neuronal differentiation, and Calcein/PI assays were conducted at day 14. An increasing trend in cell viability was observed in both WT-iMNs and P497H-iMNs (Figure 7A,B and Figure S4), although the differences were not statistically significant (WT-DMSO vs. WT-CHX *p* = 0.11; P497H-DMSO vs. P497H-CHX *p* = 0.34).

### 2.5. Bosutinib Resists Oxidative Stress and Rescues Neuronal Death

The broad inhibitory effect of CHX on short-cycle proteins may explain its limited ability to rescue neuronal death. Targeted interventions that focus on the specific oxidative stress pathways influenced by CHX could yield more promising results. Autophagy plays a crucial role in clearing abnormal protein accumulation under oxidative stress, a process particularly relevant in neurodegenerative diseases. UBQLN2 mutations have been extensively reported to cause autophagy impairment and the accumulation of ubiquitinated proteins in various models. To verify this, we performed Western blot analysis on iMN lysates, showing that p62 and LC3 levels were significantly higher in P497H-iMNs compared to WT-iMNs (Figure S5). CHX was able to mitigate oxidative stress-induced p62 accumulation (Figure 5D,E), and ROS levels were elevated in P497H-iMNs (Figure 3C).

Based on above findings, we propose that enhancing autophagy may improve cell viability in P497H-iMNs. Imamura et al. found that the Src/c-Abl inhibitor bosutinib enhances autophagy, reducing the accumulation of misfolded SOD1 proteins, which benefits the survival of iPSC-derived motor neurons from sporadic ALS and familial ALS associated with TDP43 and C9orf72 [46].

To confirm whether bosutinib also benefits P497H-iMNs survival, we differentiated motor neurons in a 12-well plate, switching to a bosutinib-containing medium on day 7 of differentiation. On day 14, we assessed cell survival using Calcein/PI staining and found that bosutinib effectively promoted the survival of P497H-iMNs, increasing relative viability by 22.3% (from 0.481 to 0.587) (Figure 7A,B). This result is consistent with reports of bosutinib in other ALS mutant motor neurons [46]. Interestingly, bosutinib was also effective in preserving cell viability in WT-iMN (Figure 7B and Figure S4).

### 2.6. CHX and Bosutinib Alleviate Ubiquitinated Protein Accumulation in iMN

**Figure 8:**
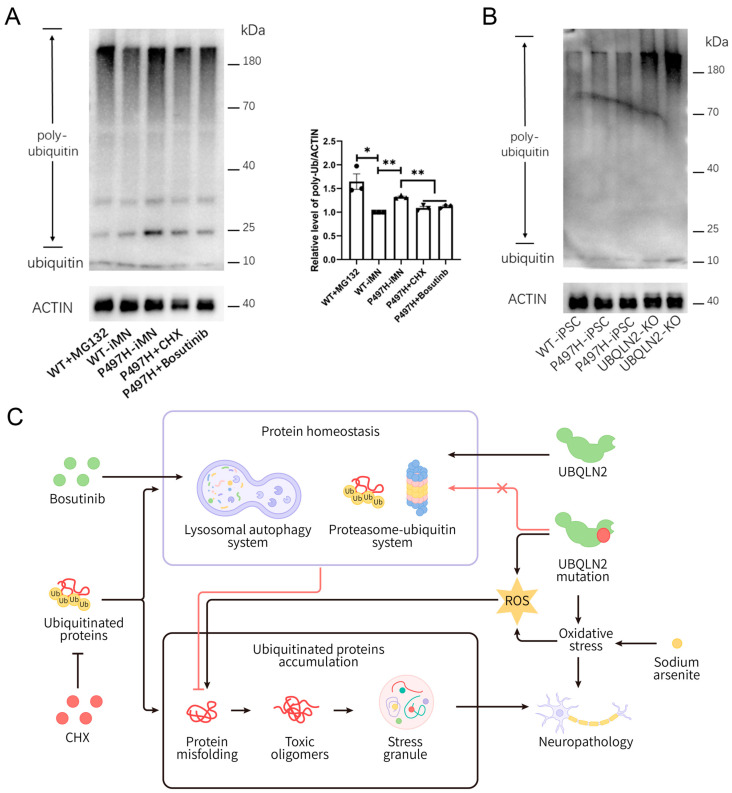
CHX and bosutinib alleviate ubiquitinated protein accumulation in iMN. (**A**) Western blot analysis of iMNs (*n* = 3). iMNs at day 7 were treated with 1 μM CHX or 1 μM bosutinib for 24 h, or 10 μM MG132 for 2 h. Data are means ± SEM. * *p* < 0.05, ** *p* < 0.01. Statistics by Student’s *t* test and one-way ANOVA. (**B**) Western blot analysis of iPSCs. The WT-iPSC and P497H-iPSC lines were derived as described earlier. The UBQLN2-KO line was generated by knocking out UBQLN2 in WT-iPSCs using CRISPR technology, and the clones were validated by sequencing and Western blot analysis. (**C**) Potential pathways of UBQLN2 and oxidative stress involvement in neuropathology.

Given the critical role of UBQLN2 in regulating protein homeostasis through autophagy and the ubiquitin-proteasome system, we performed Western blot analysis to assess ubiquitinated protein levels in iMNs (Figure 8A). The results showed that P497H iMNs exhibited significantly elevated ubiquitinated protein levels compared to WT iMNs. As a positive control, treatment with MG132 confirmed proteasomal system impairment in P497H motor neurons. Importantly, CHX and bosutinib both reduced ubiquitinated protein levels in P497H iMNs.

To further elucidate the impact of the P497H mutation, we conducted additional analyses using UBQLN2-KO iPSCs (Figure 8B). Compared to WT, UBQLN2-KO cells exhibited notable ubiquitinated protein accumulation. Our experiments support the hypothesis that the UBQLN2 P497H mutation contributes to ubiquitinated protein accumulation via a loss-of-function mechanism, particularly in motor neurons. Both CHX and bosutinib effectively reduced ubiquitinated protein levels, ameliorating neuronal phenotypes.

## 3. Discussion

In this study, we established the UBQLN2 497H NIL-iMN model, which effectively recapitulates several ALS neuropathological phenotypes, including TDP-43 mislocalization, axonal damage, neuronal degeneration, increased LDH release, abnormal SG formation, and increased ROS production. The iPSC-iMN model is a well-established in vitro system for studying neurodegeneration and has been widely used for investigating neuronal phenotypes, mechanisms, and drug screening [36,37,46,47]. The UBQLN2 497H NIL-iMN model can be utilized for high-throughput drug screening to identify compounds that mitigate the toxic effects of UBQLN2 mutations, thereby accelerating the discovery of potential ALS therapies. This platform provides a means to study the complex mechanisms underlying protein homeostasis, stress response pathways, and neuronal degeneration in ALS.

Protein misfolding and oxidative stress are key pathogenic mechanisms in neurodegenerative diseases (ND), yet their connection in UBQLN2 ALS has been underexplored [6,13,15,16,19,24]. One possible mechanism is that UBQLN2, through its UBA domain, interacts with ubiquitinated proteins, facilitating the delivery of misfolded proteins to the proteasome for degradation (Figure 8C). Mutations impair this delivery, leading to the accumulation of misfolded proteins [24]. These misfolded proteins can disrupt mitochondrial integrity, increasing ROS production and causing oxidative stress. Oxidative stress phosphorylates eIF2α, inhibits translation, disrupts cellular protein homeostasis, and further accelerates abnormal protein aggregation. Another possible mechanism is that oxidative stress, through oxidative protein damage, promotes the accumulation and aggregation of ubiquitinated proteins, influencing UBQLN2 intracellular aggregation and contributing to ALS pathology. Our results indicate that CHX and bosutinib ameliorated abnormal protein accumulation in iMNs.

Stress granule (SG) studies are widely used in ND research to investigate abnormal protein aggregation [32,33,48]. SA is a classic inducer of oxidative stress and SG formation, including acute oxidative stress induced by high concentrations (500 µM) and chronic oxidative stress by low concentrations (1–50 µM) [32,49]. SGs are thought to share regulatory pathways with abnormal protein aggregation in neurodegenerative diseases [32,33,48,50]. Recent studies reported that SGs colocalize with TDP-43 in ALS motor neurons under chronic oxidative stress [32]. We observed a similar phenomenon in UBQLN2-related ALS motor neurons, further supporting that SGs can partially represent abnormal protein aggregation in ALS [31]. Persistent SGs formed under chronic oxidative stress resemble abnormal protein accumulation in NDs, which is detrimental to cell survival. Oxidative stress is linked to various pathways in neurodegenerative diseases, including abnormal protein accumulation, DNA damage, and mitochondrial dysfunction [9,12,14]. We found that chronic oxidative stress affects UBQLN2 ALS neuronal pathology through abnormal protein accumulation pathways.

Our study shows that CHX can inhibit SG formation under chronic oxidative stress and rescue oxidative stress-related neuronal axonal degeneration. Although CHX generally inhibits protein synthesis and can be harmful under normal physiological conditions, it demonstrates protective effects in stress scenarios [35,51,52,53]. Our study is the first to evaluate CHX’s protective effects against SA-induced oxidative stress in iMNs and HeLa cells. CHX protected cells from SA-induced SG formation, morphological changes, axonal degeneration, apoptosis, and necrosis. However, the mechanism by which CHX rescues cell death remains unclear. One explanation is that CHX inhibits the production of proteins related to programmed cell death, thereby interrupting the cell death process. However, the specific molecules involved vary, with heat shock proteins and p53 reported to play roles [51,54]. Recent studies suggest that CHX may exert anti-apoptotic effects by inhibiting SG formation and the sequestration of the key apoptotic protein ZBP1 [35]. We report that CHX rescues the accumulation of oxidative stress-related p-eIF2a, p62, G3BP1, and ubiquitinated proteins. Although CHX demonstrated the ability to inhibit SG formation and rescue cell death under oxidative stress, its nonspecific cytotoxicity makes it unsuitable for clinical applications. Identifying the specific mechanisms and key molecules by which CHX rescues oxidative stress-related neuronal pathology may benefit research into alternative compounds with more specific and safer profiles for clinical use.

Bosutinib has shown promise in regulating autophagy and rescuing neuronal death, particularly in the context of neurodegenerative diseases like ALS [46,55,56]. Autophagy dysfunction, commonly observed in ALS, contributes to the accumulation of misfolded proteins and neuronal degeneration [50,57,58,59]. Studies have indicated that bosutinib, a Src/c-Abl inhibitor, enhances autophagy and reduces the accumulation of misfolded proteins, such as mutant SOD1, which are implicated in ALS pathology [46]. A phase I clinical trial involving 13 patients treated with bosutinib has been reported, in which some patients responded well to the treatment and maintained clinical stability over 12 weeks [56]. Notably, among two patients with SOD1 mutations, one exhibited stable ALSFRS-R scores after treatment with 100 mg, while the other discontinued treatment due to adverse effects. Due to the small sample size and the heterogeneity in disease progression among ALS patients, the efficacy of bosutinib cannot be conclusively determined at this time. We report the neuroprotective effects of bosutinib on the survival of UBQLN2 mutant ALS iMNs, highlighting its potential mitigating neuronal death in UBQLN2-related ALS. Given the uncertain clinical efficacy of bosutinib, further research is needed to confirm its therapeutic potential in UBQLN2-related ALS.

We used a common treatment-evaluation scheme, treating on day 7 post-differentiation and assessing on day 14. However, the effects of CHX and bosutinib in neurons were suboptimal. One possible explanation is that motor neurons are non-regenerative, and early cell damage may be difficult to rescue with later drug treatments.

Our study is limited by using only a few drugs to rescue UBQLN2 497H-iMN neuropathology, sourced from existing literature without a high-throughput drug screen targeting oxidative stress pathways. Additionally, the rescue effects of these drugs on neuronal pathology were suboptimal. On the one hand, as non-dividing cells, neurons lack the cell division needed for cellular remodeling, making their pathological damage difficult to reverse [60]. On the other hand, the complexity of ALS pathogenesis, with numerous causative genes, means many reported drugs show inconsistent effects across different models [46,61]. Exploring ALS pathogenesis and finding more effective ALS therapeutic drugs remain key challenges in ALS research.

In conclusion, we found that oxidative stress involved in SG-related abnormal protein aggregation contributes to axonal degeneration and cell death in ALS neurons and HeLa cells. CHX and bosutinib can rescue oxidative stress-related neuronal degeneration and cell death to varying degrees. Our research highlights the critical involvement of abnormal protein aggregation in the neurodegenerative pathology of UBQLN2-associated ALS motor neurons under oxidative stress conditions. The findings suggest that oxidative stress exacerbates protein misfolding and aggregation, contributing to neuronal damage and disease progression in this ALS model. Understanding this interaction between oxidative stress and protein aggregation could provide new insights into therapeutic strategies for ALS and other neurodegenerative disorders.

## 4. Materials and Methods

### 4.1. Materials

The cell culture materials were sourced from Gibco (Waltham, MA, USA) and included DMEM/F12, DMEM, FBS, NeuralBasal medium, dispase, B27 supplement (50×), N2 supplement (100×), and 1× GlutaMAX (100×). mTesR Plus was obtained from Stemcell Technologies (Vancouver, BC, Canada). Matrigel matrix was purchased from Corning. The following compounds were acquired from Selleck (Houston, TX, USA): DMH1, CHIR99021, SB431542, purmorphamine (Pur), DAPT, Y27632, MG132, bosutinib, and cycloheximide (CHX). Sodium arsenite, retinoic acid (RA), valproic acid, and doxycycline (Dox) were purchased from Sigma-Aldrich (St. Louis, MO, USA). Compound E was obtained from Yeasen (Shanghai, China). The ATP cell viability assay kit, Calcein/PI cell viability and cytotoxicity detection kit, LysoTracker Red, and Hoechst were sourced from Beyotime (Shanghai, China). The LDH assay kit was purchased from the Nanjing Jiancheng Bioengineering Institute (Nanjing, China).

The plasmid CLYBL-TO-hNIL-BSD-mApple was generously provided by Michael Ward (Addgene plasmid #124230). The plasmid px330-CLYBL was generated by introducing the sgRNA sequence targeting the CLYBL safe harbor site into px330 after digestion with BbsⅠ (NEB, Ipswich, MA, USA). The CLYBL sgRNA cleavage site is compatible with the plasmid CLYBL-TO-hNIL-BSD-mApple. The CLYBL safe harbor sgRNA sequence used was ATGTTGGAAGGATGAGGAAA.

### 4.2. Cell Culture

All cells were routinely cultured at 37 °C in a humidified incubator with 5% CO_2_.

Healthy control iPSCs and UBQLN2 P497H iPSCs were generated and have been previously reported [31,62]. iPSCs were cultured in mTeSR Plus medium on Matrigel-coated plates.

HeLa cells, maintained in our laboratory and originally obtained from ATCC, were cultured in Dulbecco’s Modified Eagle Medium (DMEM) supplemented with 10% fetal bovine serum (FBS). The G3BP1-GFP HeLa cell line was generated by linking the GFP sequence to the 3′ end of the final exon of G3BP1 using the CRISPR/Cas9 system.

### 4.3. Construction of NIL-iPSCs

NIL-iPSCs were generated by introducing the NIL conditional expression elements into the genomes of both control and UBQLN2 mutant iPSCs. Specifically, the px330-CLYBL plasmid and the CLYBL-TO-hNIL-BSD-mApple plasmid were introduced into iPSCs using the Human Stem Cell Nucleofector Kit 2 (Lonza, Basel, Switzerland, VPH-5022). G418 selection was employed to isolate clones, which were then identified by PCR and sequencing. Flow cytometry was used to assess mApple signal to determine clone purity, with clones where over 90% of cells were mApple-positive considered satisfactory. Off-target predictions were performed using CCTop—CRISPR/Cas9 target online predictor, and Sanger sequencing was conducted on NIL-iPSCs to verify off-target effects. Clones were considered satisfactory if no mutations were detected at the top ten predicted off-target sites. If necessary, CRE-GFP plasmid transfection was performed to remove selection markers flanked by loxP sites in NIL-iPSCs. Through this process, we obtained healthy control WT-NIL-iPSCs and UBQLN2 P497H NIL-iPSCs.

### 4.4. Induced Motor Neuron Differentiation

The differentiation protocol for NIL-iMNs was adapted from a previous report [63]. Briefly, NIL-iPSCs were seeded in 6-well plates and induced to differentiate using induction medium (IM) containing 3 µg/mL of the inducer the following day. After 2 days, cells were digested with dispase and replated at an appropriate density on Matrigel-coated plates. They were then cultured for one day in IM supplemented with Y27632 and BrdU to remove proliferating cells. Following this, cells were cultured in maturation medium (MM) for 7 days to obtain mature motor neurons. Motor neuron markers were identified using immunofluorescence and RT-qPCR. The compositions of IM and MM can be obtained from the previous report [63].

### 4.5. RT-qPCR Identification of Neuronal Markers

RNA was extracted from motor neurons using Trizol, and reverse transcribed into cDNA with a reverse transcription kit (Vazyme, Nanjing, China, R223). The expression levels of HB9 and CHAT were assessed using a qPCR kit (Vazyme, Q711), with GAPDH as the internal control. The corresponding primers are as follows:

qHB9-F1: CTCCTACTCGTACCCGCAG

qHB9-R1: TTGAAGTCGGGCATCTTAGGC

qCHAT-F1: CAGCCCTGCCGTGATCTTT

qCHAT-R1: TGTAGCTGAGTACACCAGAGATG

### 4.6. Immunofluorescence

Cells were fixed at room temperature with 4% paraformaldehyde for 15 min. Following fixation, cells were permeabilized with 0.2% Triton X-100 for 10 min and then blocked with 5% BSA in PBS for 1 h. Fixed cells were incubated with the following primary antibodies: anti-G3BP1 (Abcam, Cambridge, UK, ab56574), anti-TDP43 (Abcam, ab109535), anti-HB9 (Merck, Darmstadt, Germany, ABN174), anti-CHAT (Merck, AB144P), and anti-SMI32 (BioLegend, San Diego, CA, USA, 801701). Incubation was performed at room temperature for 2 h or overnight at 4 °C, followed by incubation with fluorescent secondary antibodies for 1 h. Nuclei were stained with 4′,6-diamidino-2-phenylindole (DAPI, Beyotime, Shanghai, China), and visualization was conducted using a fluorescence microscope (Zeiss, Oberkochen, Germany) or a confocal microscope (Zeiss LSM 980).

### 4.7. Western Blot Analysis and Antibodies

Cells were lysed using RIPA buffer (Beyotime). Proteins were separated by 10% or 12.5% SDS-PAGE and then transferred to PVDF membranes (Millipore, Burlington, MA, USA). Western blotting was performed using the following primary antibodies: anti-p-eIF2α (Cell Signaling Technology, Danvers, MA, USA, 9721), anti-eIF2α (Cell Signaling Technology, 9722), anti-p-mTOR (Cell Signaling Technology, 5536), anti-mTOR (Cell Signaling Technology, 2983), anti-p62 (Abcam, ab109012), anti-UBQLN2 (Abnova, Taipei, Taiwan, H00029978-M03), anti-Ubiquitin (Diagbio, Hangzhou, China, db11104), anti-LC3A/B (Abcam, ab62721), and anti-Bax (Santa Cruz Biotechnology, Dallas, TX, USA, sc7480). Protein bands were visualized using an ECL detection kit (Thermo Fisher Scientific, Waltham, MA, USA, 34080).

### 4.8. Cell Viability Analysis

Cell viability of NIL-iMNs was assessed using an ATP cell viability detection kit. Briefly, NIL-iPSCs were seeded at a density of 10^4^ cells/mL in 96-well plates for differentiation. ATP levels were measured on days 7 and 14 of differentiation to compare the ATP decrease between normal and ALS motor neurons, thus evaluating relative cell viability.

Cell viability and cytotoxicity of HeLa cells and NIL-iMNs were also assessed using the Calcein/PI cell viability and cytotoxicity detection kit (Beyotime, C2015). Visualization was performed using a Zeiss fluorescence microscope and a live-cell light-sheet imaging microscope (Zeiss/Axio Observer 7). Calcein/PI signal ratios were analyzed using ImageJ to determine cell viability.

### 4.9. ROS Detection

To assay the ROS level, a commercial ROS assay kit (Beyotime, S0033) was used and operated as described in the protocols. To conduct the experiment, iMNs were seeded into 96-well plates and cultured until they matured into motor neurons (day 14). Fresh medium was added, and 10 µL of fluorescent dye DCFH-DA was incubated in each well at 37 °C for 30 min. Fluorescence intensity in each well was then measured using a fluorescent plate reader. For fluorescence detection, the excitation wavelength was at 488 nm, and the emission peak was examined at 525 nm. After subtracting the background control, the results were normalized using ATP levels from parallel wells to account for any effects related to cell number.

### 4.10. Axonal Transport Analysis

Final motor neuron differentiation was conducted in confocal dishes as described previously [31]. Motor neurons from both UBQLN2 mutant and control groups (day 7) were stained with LysoTracker-Red (50 nM). After washing, the neurons were equilibrated in motor neuron maturation medium for 20 min. Motor neurons were selected based on their typical morphology of cell body and long extending axons under a live-cell light-sheet imaging microscope. LysoTracker-Red was excited at 580 nm, and image sequences (3 s/frame, total of 2 min) were captured using a cooled CCD camera (Zeiss/Axio Observer 7) with Zen Pro (Zeiss Zen 3.7) software.

All analyses were conducted as described before [64], using the KymographClear plugin in Fiji (ImageJ). Briefly, kymographs or space-time plots were generated for each neuronal process. In these plots, stationary lysosomes appear as vertical lines, while moving lysosomes produce inclined lines. The proportion of moving versus stationary lysosomes was extracted by annotating and analyzing the trajectory properties of each inclusion body.

### 4.11. Statistical Analysis

Flow cytometry analysis data were analyzed using FlowJo (v10). Immunoblot density measurements, cell counts, SG counts, and neurite tracking were performed using Fiji (ImageJ 1.54). Data were collected and graphed using Prism 8.0 (GraphPad Software). Statistical comparisons between two groups were performed using Student’s t-test. For comparisons among two or more groups, one-way or two-way analysis of variance (ANOVA) was used, followed by Tukey’s or Sidak’s multiple comparison tests. Statistical significance was determined as * *p* < 0.05, ** *p* < 0.01, *** *p* < 0.001, **** *p* < 0.0001.

## 5. Conclusions

This study established the P497H-NIL-iMN model based on P497H-iPSC, which exhibits ALS-characteristic TDP43 cytoplasmic mislocalization, along with neurodegenerative phenotypes such as autophagy abnormalities, axonal degeneration, and increased apoptosis. The P497H-iMN model enriches the repertoire of UBQLN2 ALS research models and provides a valuable tool for investigating pathogenic mechanisms. Our study demonstrates that chronic oxidative stress may contribute to the neurodegeneration observed in UBQLN2 mutant ALS motor neurons by promoting abnormal protein aggregation, which in turn leads to axonal swelling, impaired axoplasmic transport, and neuronal degeneration. Importantly, inhibition of abnormal protein aggregation can rescue neuronal pathological phenotypes to varying degrees. These findings suggest that chronic oxidative stress drives axonal pathology in UBQLN2 P497H ALS motor neurons through abnormal protein aggregates, offering valuable insights into the roles of oxidative stress and protein aggregation in ALS.

## Data Availability

The original contributions presented in the study are included in the article/Supplementary Material; further inquiries can be directed to the corresponding authors.

## Supplementary Materials

The following supporting information can be downloaded at: https://www.mdpi.com/article/10.3390/ijms252413448/s1.
