# Supplementary material for: Chronic Oxidative Stress and Stress Granule Formation in UBQLN2 ALS Neurons: Insights into Neuronal Degeneration and Potential Therapeutic Targets
Source: Int J Mol Sci. 2024 Dec 15;25(24):13448. doi: 10.3390/ijms252413448 (PMC11678478; doi:10.3390/ijms252413448)
Supplement: Supplementary file 1 [file ijms-25-13448-s001.zip › Supplement.pdf]

# Figure S1

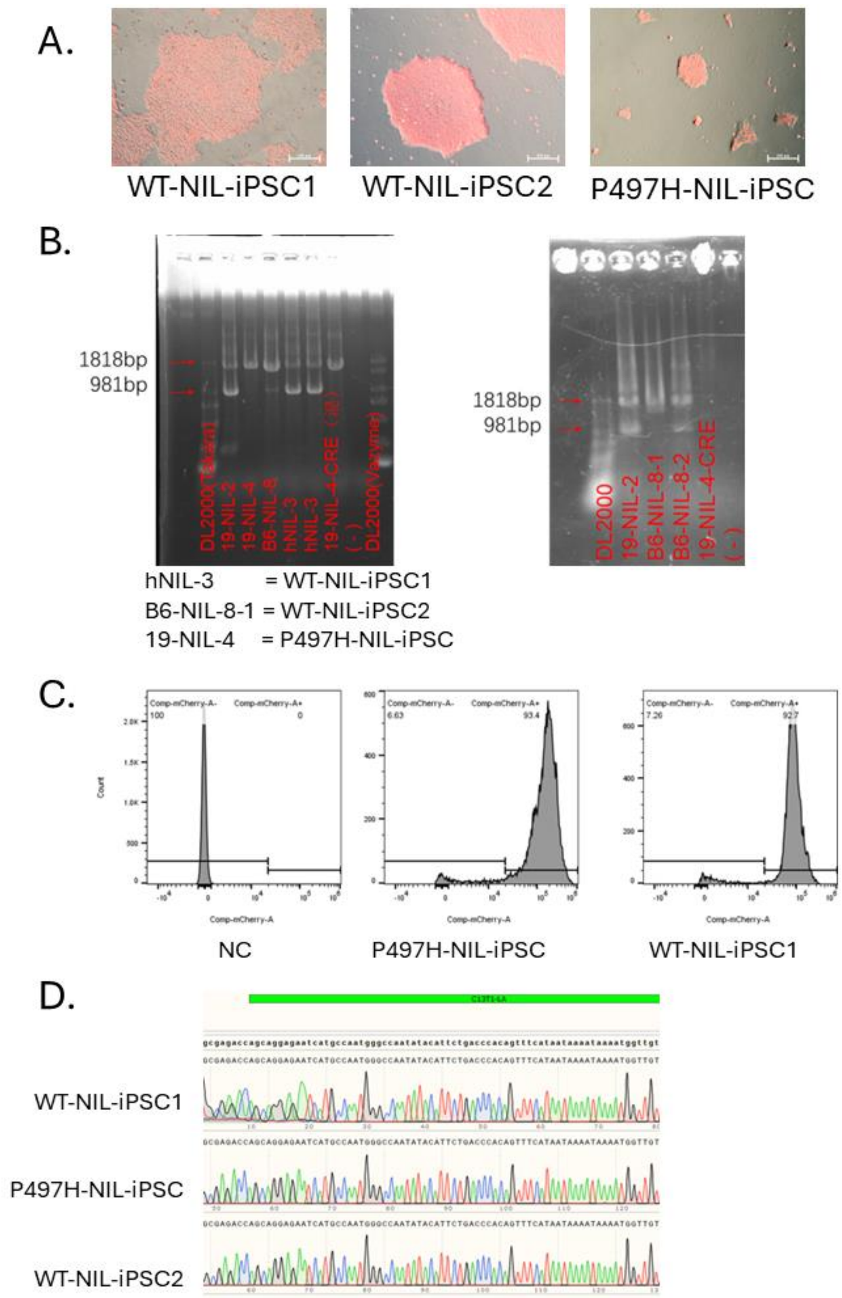


**Figure S1. Characterization of NIL-iPSCs.** (A) Fluorescence identification: The CRISPR/Cas9 gene-editing donor vector carries the mApple fluorescent marker gene. Fluorescence microscopy confirmed successful vector integration. (B) PCR-based site-specific integration identification: A pair of primers was designed across the CLYBL homology arms, combined with one downstream primer on the vector, to establish a three-primer PCR system. The presence of an 1818 bp product indicates successful site-specific integration, while the 981 bp product indicates the presence of unedited CLYBL alleles. Results show that clone 19-NIL-4 and clone B6-NIL-8-1 lack the 981 bp product, suggesting that these two clones are homozygous with sufficient purity. (C) Flow cytometry analysis of selected clones: Clone hNIL-3, identified by PCR as potentially heterozygous, was further analyzed by flow cytometry to determine clone purity. The percentage of mApple-positive cells was measured, with 19-NIL-4 serving as a positive control. Results indicate that hNIL-3 has a 92.7% mApple-positive rate, confirming adequate purity. (D) Sequencing verification of gene-editing sites in iPSCs: PCR amplification across the homology arms followed by sequencing confirmed the targeted integration of the donor vector at the CLYBL locus.

# Table S1

| Coordinates | MM | target_seq | PAM | distance | | gene name |
|---|---|---|---|---|---|---|
| chr13:99822975-99822997 | 0 | ATGTTGGA [AGGATGAGGAAA] | TGG | 35864 | I | CLYBL |
| chr17:4986383-4986405 | 2 | ATCTGGGA [AGGATGAGGAAA] | GGG | 61 | I | RP5-1050D4.4 |
| chr8:102671574-102671596 | 3 | AAAGTGGA [AGGATGAGGAAA] | AGG | 15672 | - | KLF10 |
| chr12:89953404-89953426 | 3 | CTGATTGA [AGGATGAGGAAA] | GGG | 3678 | I | RP11-654D12.3 |
| chr20:19569402-19569424 | 4 | GGAGTGGA [AGGATGAGGAAA] | GGG | 10576 | I | SLC24A3 |
| chr4:170133498-170133520 | 4 | GGGGAGGA [AGGATGAGGAAA] | AGG | 7772 | - | RP11-654F3.1 |
| chr5:83058716-83058738 | 4 | TAATTTGA [AGGATGAGGAAA] | AGG | 1562 | I | TMEM167A |
| chr11:65698829-65698851 | 3 | ATTATGAA [AGGATGAGGAAA] | GGG | 2992 | - | RN7SL309P |
| chr2:162785013-162785035 | 4 | GGGTATGA [AGGATGAGGAAA] | AGG | 0 | E | AC007740.1 |
| chr15:86788055-86788077 | 4 | AAAGTAGA [AGGATGAGGAAA] | TGG | NA | I | NA |

**Table S1.** CLYBL sgRNA off-target prediction using CCTop - CRISPR/Cas9 Target Online Predictor. The table lists the top ten predicted off-target binding sites. Primers were designed for these off-target sites, and sequencing analysis was performed following PCR amplification. No off-target mutations were detected in any of the iPSC lines involved in our study.

# Figure S2

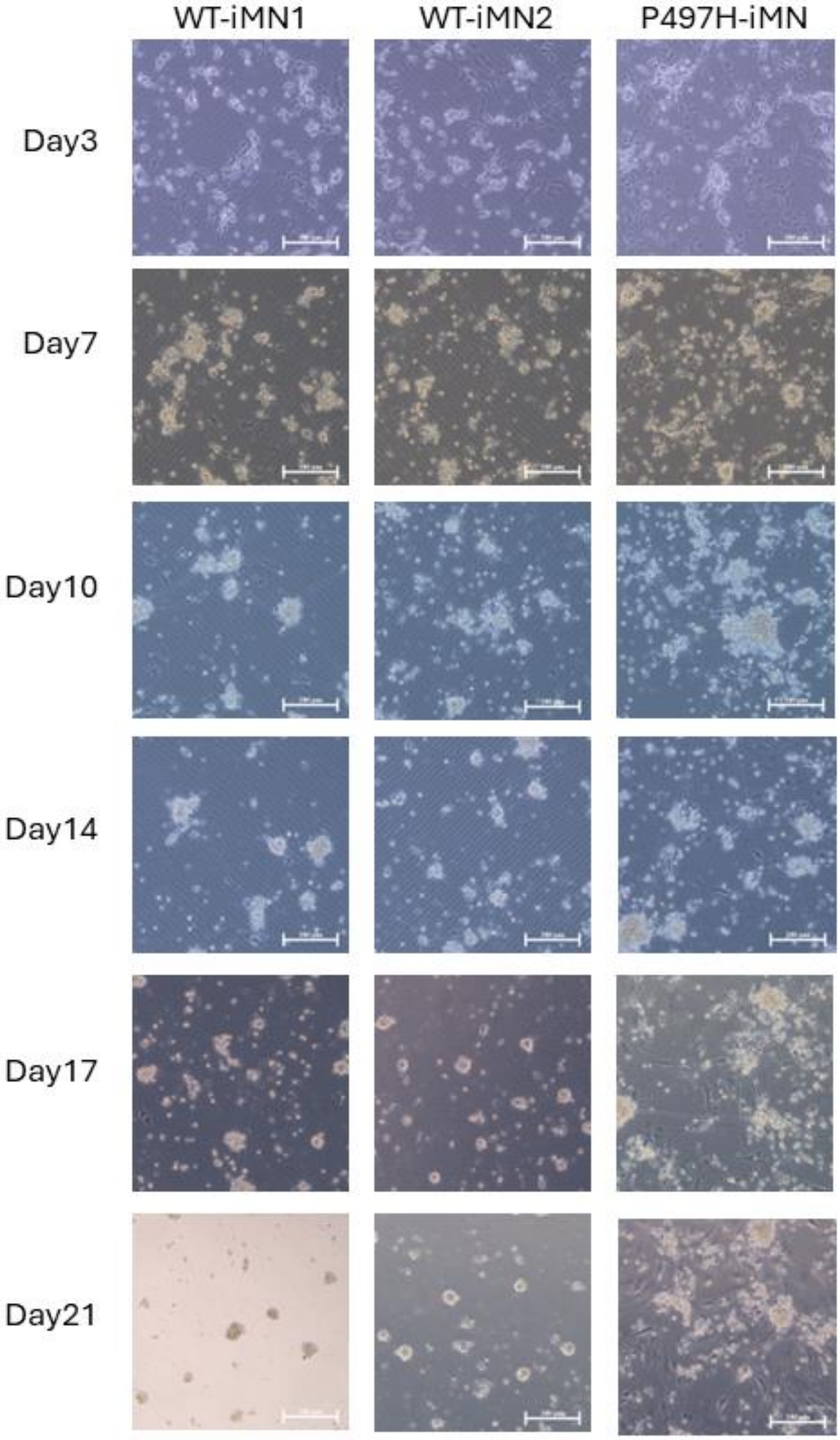


**Figure S2.** Cell morphology of NIL-iMN differentiation from Day 3 to Day 21. The results show that the P497H-iMN cell body clusters gradually enlarge during the differentiation process and long-term culture.

# Figure S3

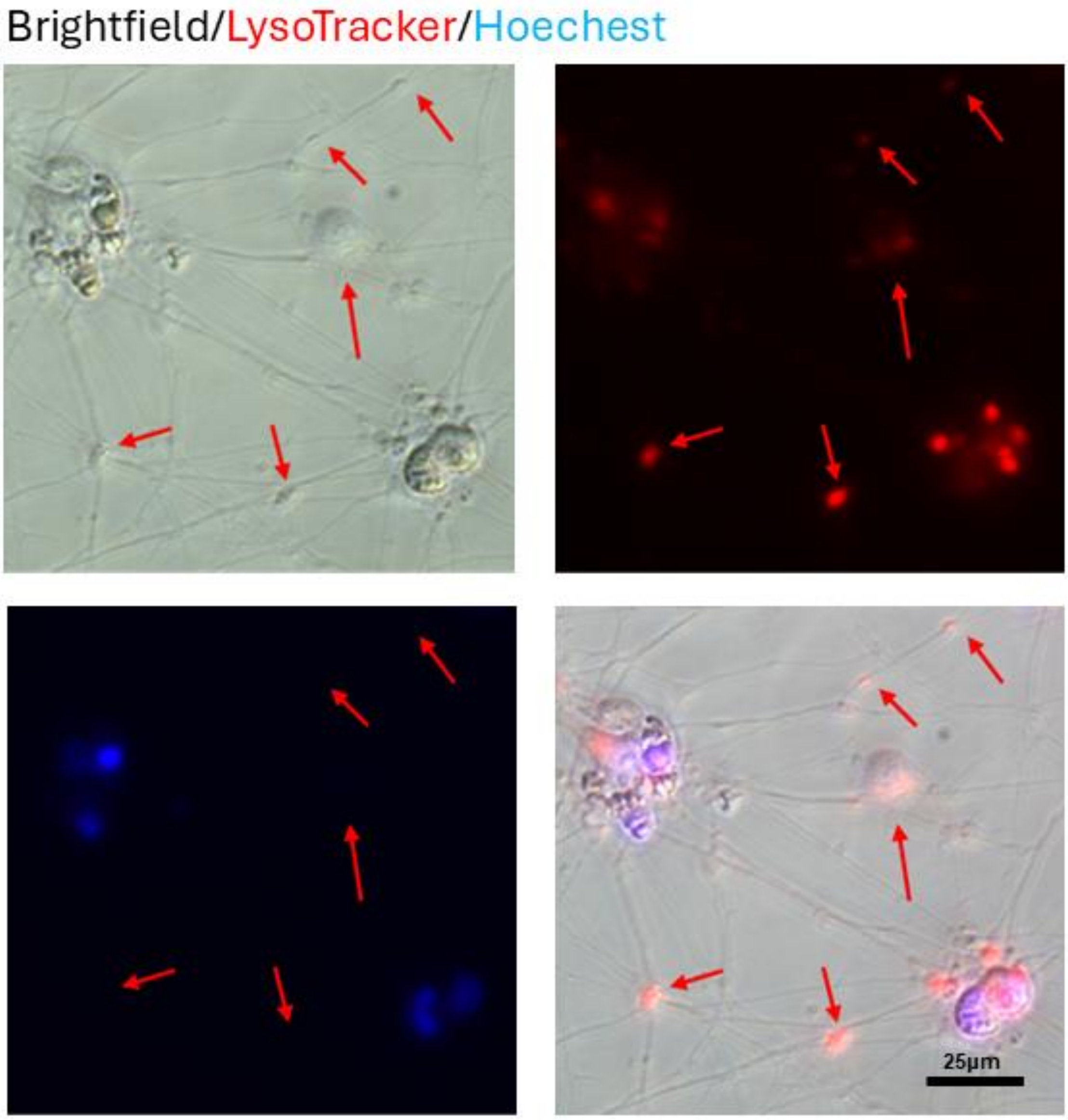


**Figure S3.** The relationship between lysosomal marker LysoTracker and axonal swelling. Fluorescence microscopy imaging of induced motor neurons revealed that LysoTracker-labeled lysosomes were distributed the neuronal soma and neurites. Axonal swelling vesicles showed positive LysoTracker staining (indicated by arrows), while Hoechst staining marked the location of the neuronal soma.

# Figure S4

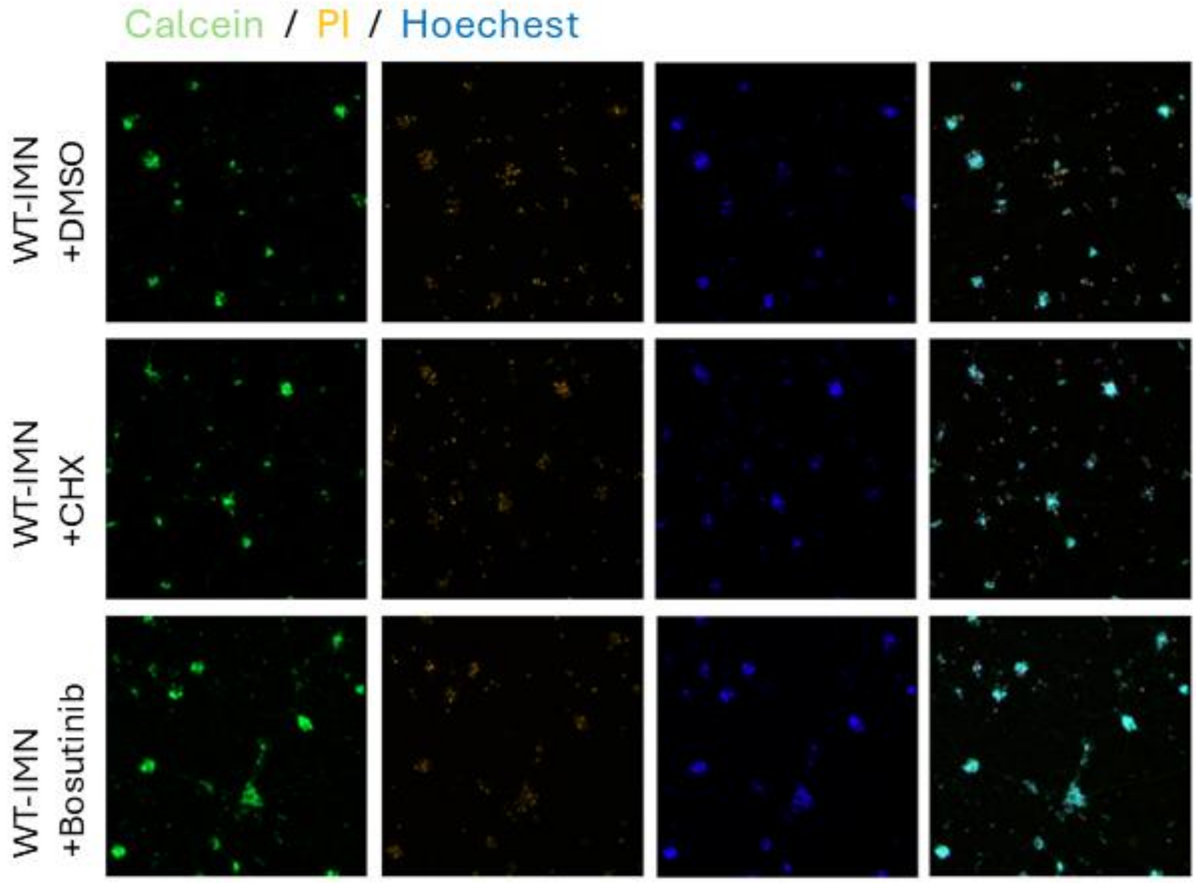


**Figure S4.** Effects of CHX and Bosutinib on Cell Viability. WT-iMNs at Day 7 were treated with 1 μM CHX and 1 μM Bosutinib, and Calcein/PI analysis was performed and imaged at Day 14.

# Figure S5

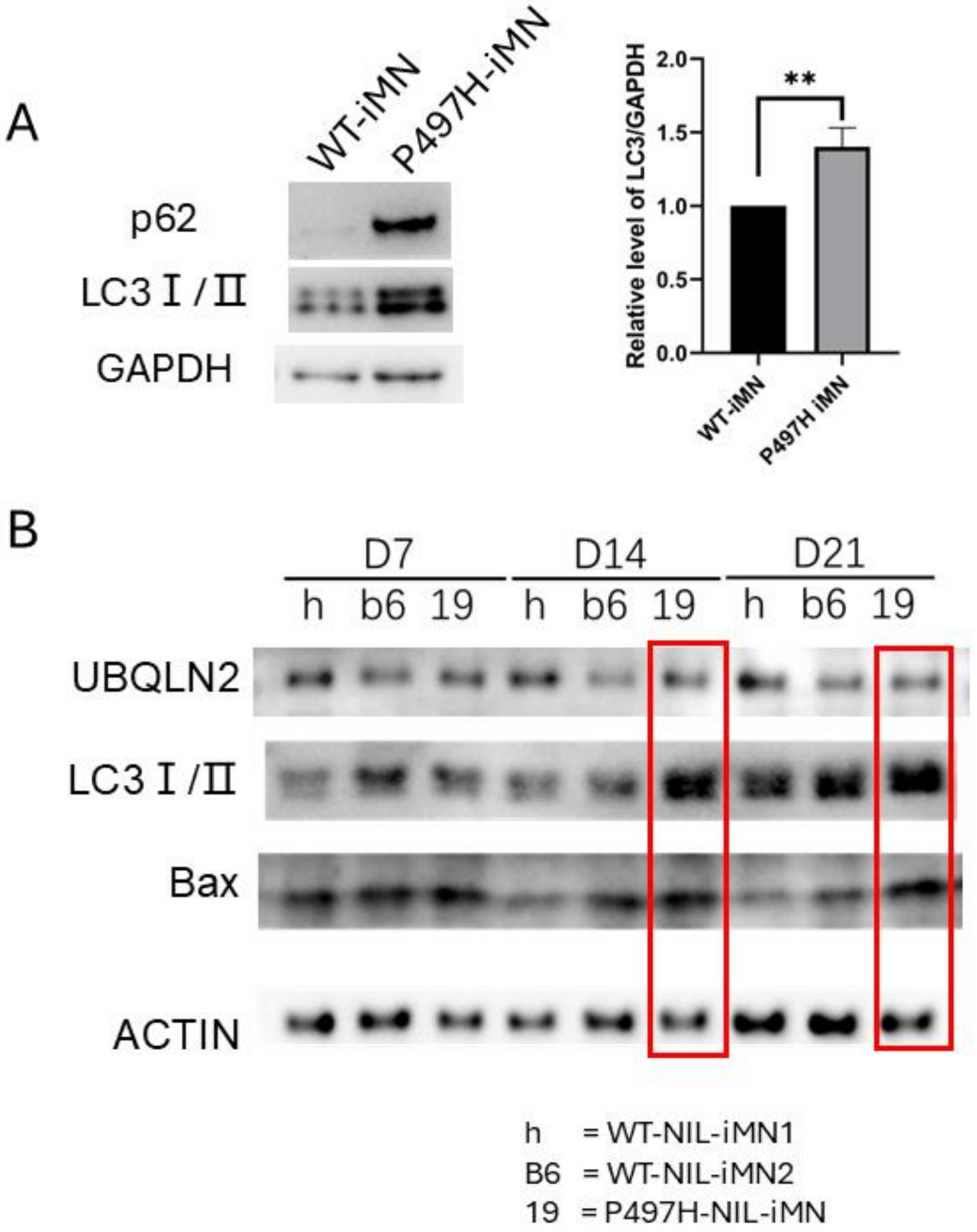


**Figure S5.** WB Analysis of Autophagy-Related Proteins in iMNs. (A) Western blot analysis of Day 14 lysates from WT-iMN and P497H-iMN reveals significantly elevated levels of p62 and LC3 in P497H-iMN, indicating impaired autophagic flux. (B) Western blot analysis at different time points shows a general increase in LC3 levels over time, with a more pronounced rise in P497H-iMN. Additionally, the apoptosis marker Bax is higher in P497H-iMN at Day 14 and Day 21 compared to controls.
